# Echolocation of static and moving objects in two-dimensional space using bat-like frequency-modulation sound

**DOI:** 10.3389/fphys.2013.00149

**Published:** 2013-07-02

**Authors:** Ikuo Matsuo

**Affiliations:** ^1^Department of Information Science, Tohoku Gakuin UniversitySendai, Japan; ^2^Neurosensing and Bionavigation Research Center, Doshisha UniversityKyotanabe, Kyoto, Japan

**Keywords:** bat, echolocation, model, localization, linear period modulation

## Abstract

Bats use frequency-modulated echolocation to identify and capture moving objects in real three-dimensional space. The big brown bat, *Eptesicus fuscus*, emits linear period modulation sound, and is capable of locating static objects with a range accuracy of less than 1 μs. A previously introduced model can estimate ranges of multiple, static objects using linear frequency modulation (LFM) sound and Gaussian chirplets with a carrier frequency compatible with bat emission sweep rates. The delay time for a single object was estimated with an accuracy of about 1.3 μs by measuring the echo at a low signal-to-noise ratio. This model could estimate the location of each moving object in two-dimensional space. In this study, the linear period modulation sounds, mimicking the emitting pulse of big brown bats, were introduced as the emitted signals. Echoes were measured from moving objects at two receiving points by intermittently emitting these sounds. It was clarified that this model could localize moving objects in two-dimensional space by accurately estimating the object ranges.

## Introduction

Bats emit high-frequency sound waves, allowing them to track and catch flying insects (Griffin, 1958; Simmons et al., 1995). Bats perceive the location of moving objects in three-dimensional (3D) space using frequency modulation. Experimental evidence indicates that bats are capable of locating static objects at high signal-to-noise ratios (SNRs) achieving sub-microsecond accuracy (Simmons, 1979; Menne et al., 1989; Moss and Schnitzler, 1989; Simmons et al., 1990). In echolocation, many kinds of bats, including *Eptesicus fuscus* and *Noctilio leporinus*, emit linear period modulation (LPM) sound, the instantaneous period of which increases linearly with time. It was clarified that the LPM signal is useful for the range estimation of moving objects because of its Doppler tolerance using matched filters (Altes and Titlebaum, 1970; Altes and Skinner, 1977). Several previously proposed models estimate the delay times of multiple objects from an echo spectrogram, which is computed by IIR filters or short-time Fourier transform, which corresponds to convolution of the constant-frequency (CF) carrier wave at each frequency (Saillant et al., 1993; Matsuo et al., 2001; Neretti et al., 2003). However, it is difficult to accurately determine the delay time for each object using the peak time, because the integration time of the cochlear filters is long. An echolocation model was proposed to estimate the delay times of multiple objects from the time–frequency pattern using linear frequency modulation (LFM) sound (Matsuo and Yano, 2004; Matsuo et al., 2004; Matsuo, 2011, 2013). In this model, the time–frequency pattern is computed through the convolution of Gaussian chirplet filters for which the carrier frequency agrees with the sweep rate of emission (Matsuo and Yano, 2004; Matsuo et al., 2004; Matsuo, 2011, 2013). It was demonstrated that this proposed model could estimate the range of the moving object or accurately localize the moving object in two-dimensional (2D) space using the interaural range difference (IRD), computed as the difference between the object's range at two receiving points. In addition, Gaussian chirplet filters have been proposed for LPM sounds (Guarato et al., 2011). The present study examines whether this model can localize moving objects in 2D space from echoes, which are measured from static and moving objects at two receiving points by intermittently emitting LPM sounds, corresponding to the emitting pulse of big brown bats.

## Methods

**Figure 1:**
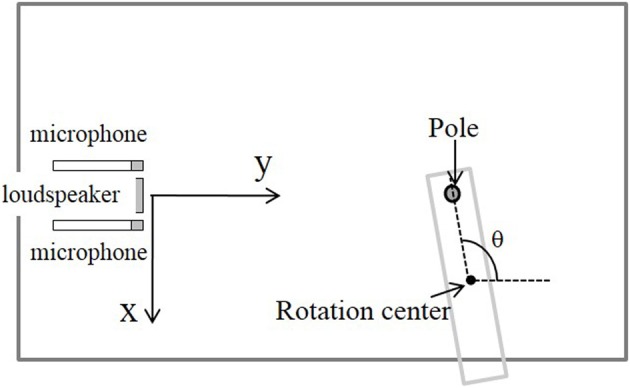
**Measuring system (top view) with a pole set on a rotating table**.

Acoustic data were recorded in a soundproof chamber (length × width × height = 2.8 m × 1.7 m × 1.8 m). The measuring system, including one loudspeaker, two microphones, and objects to be detected were located on an optical base (Chuo Precision Industrial, TT-D6090), as shown in Figure 1. The loudspeaker and microphones were placed at a height of 70 cm, and the distance between them was 4 cm. The origin was defined as the center of the speaker's surface. The reflecting objects used were erect poles (radius of 8 mm) set on a rotating table controlled by a computer via an electric rotary actuator (Taiyo, ESR1).

**Figure 2:**
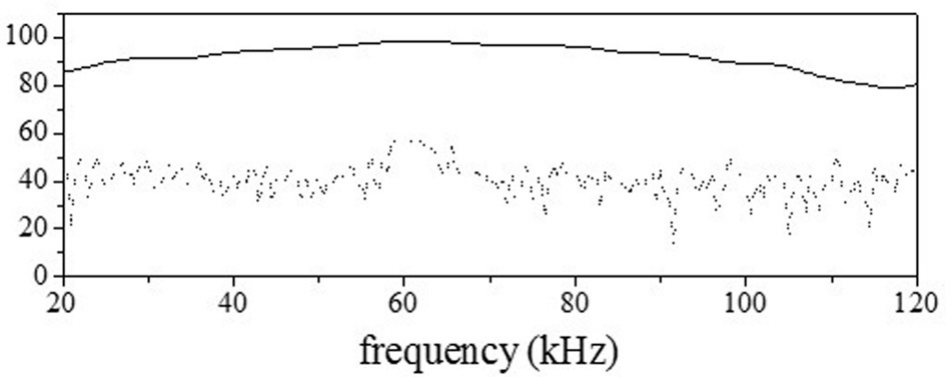
**Characteristics of the loudspeaker**. The solid curve shows the amplitude spectrum computed from the measured waveform with the loudspeaker facing the microphone. The dotted curve shows the noise level.

The emitted signal was generated by a computer (National Instruments, PXI-8106), digital-to-analog (DA) converted (PXI-5412), amplified (TDT, ED1), and emitted by the loudspeaker (TDT, ES1). The sampling frequency of the DA converter was 1 MHz, and the resolution was 16 bits. The echoes reflected by the objects were recorded using a 1/8-inch condenser microphone (Brüel & Kjær, 4138), amplified (Brüel & Kjær, NEXUS 2670, 2690), bandpass-filtered (NF, CF-4BL, CF-4BH), and analog-to-digital (AD) converted (NI, PXI-6133). The sampling frequency of the AD converter was 400 kHz, and the resolution was 14 bits. The temperature was measured in the chamber to compute the sound velocity. To estimate the characteristics of the measuring system, LFM sound, sweeping from 135 to 5 kHz over 2 ms, was used and the waveform was measured when the loudspeaker and microphone were positioned face-to-face. Figure 2 shows the spectrum computed by taking the Fourier transform of the measured waveform. At a distance of 40 cm, the maximum and average values for the amplitude spectrum in the range of 30–100 kHz were 98.6 and 89.3 decibels sound pressure level (dB SPL), respectively, and the half-power (3-dB) bandwidth was 32 kHz (Matsuo, 2013).

**Figure 3:**
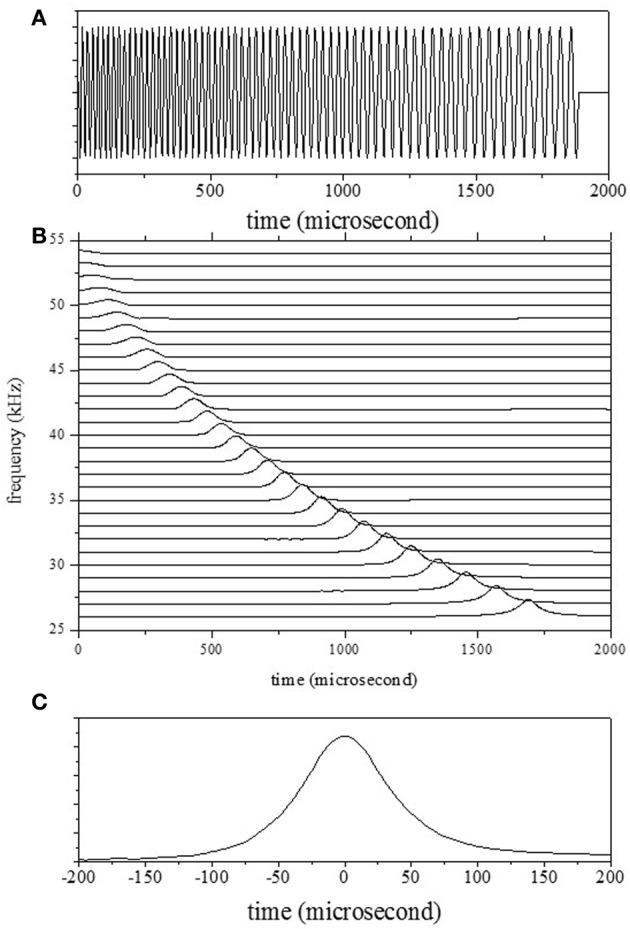
**(A)** Emission waveform. **(B)** Emission spectrogram, computed by convolution of the Gaussian chirplets. **(C)** The temporal emission pattern *W*_emi_ corresponds to the spectrogram for one bandpass filter (with a center frequency of 30 kHz).

In this paper, the bat-like LPM sound signal was synthesized by referring to the sound emitted by a big brown bat, *Eptesicus fuscus*, during approach of an object. The sound duration was almost 1.9 ms and the LPM signal started at 53 kHz and swept down to 25 kHz. Figure 3A shows the emitted waveform. The echoes were measured from the rotating pole for two situations. The first measurement was of the echo from the object moving back and forth, and the second was of the echo from the object moving from side to side. In addition, to clarify the effect of the Doppler shift on the accuracy, echoes from the static object were measured.

## Model

### Transformation of the waveform into a spectrogram using chirplet filters

The waveforms of the object echoes were entered into the echolocation model discussed in the Introduction. They were transformed into spectrograms in a manner that simulated the process in the mammalian cochlea. The temporal changes in the interference pattern were extracted using Gaussian chirplet filters with a carrier frequency consistent with the sweep rate of emission (Matsuo and Yano, 2004; Matsuo et al., 2004; Guarato et al., 2011). The temporal characteristics of the filter can be described by

(1) $$F(f_{j},t)=\exp\left(-\frac{t^{2}}{\alpha_{j}}\right)\exp\left(2\pi j\frac{\ln(kt+l)}{k}\right)$$

Here *f*_*j*_ (kHz) is the center frequency for the *j*th bandpass filter, *t* is time (s), and α_*j*_ is a parameter that describes the width of the window function:

$$\begin{array}{l} \alpha_{j}=\frac{w/2}{\ln(0.7)},\\ w=bw\left(\frac{f_{1}}{f_{j}}\right), \end{array}$$

where *f*_1_ is the start frequency of the signal and *bw* is the filter's base bandwidth fixed as 160 μs. Constants *k* and *l* are defined by

$$\begin{array}{l} k=\frac{f_{\text{sta}}-f_{\text{end}}}{\text{dur}\cdot f_{\text{sta}}\cdot f_{\text{end}}},\\ l=\frac{1-k\cdot t_{\text{sta}}\cdot f_{\text{sta}}}{f_{\text{sta}}}, \end{array}$$

where dur is the duration of the signal, *f*_sta_ (53 kHz) and *f*_end_ (25 kHz) are the starting and end frequencies of the signal, and *t*_sta_ is the start time of the signal. The bandpass filter bank comprised 24 filters with center frequencies ranging 27–50 kHz, positioned at regular intervals. The quality factor at 10 dB (Q10 dB) values ranges from 1.7 at 27 kHz to 3.1 at 50 kHz.

The waveforms for both the emitted waves and the echoes were transformed into a spectrogram *P*(*f, t*) through convolution with the filters as shown in Equation 1. Figure 3B shows the outputs of the cochlear filters for the emitted waveform. Figure 3C shows the temporal pattern corresponding to the spectrogram *P*(*f, t*) of the emission for one filter (with a center frequency of 30 kHz). The shapes of the temporal patterns corresponding to the spectrogram *P*(*f, t*) for all filters were the same because the window lengths were set dependent on center frequencies.

**Figure 4:**
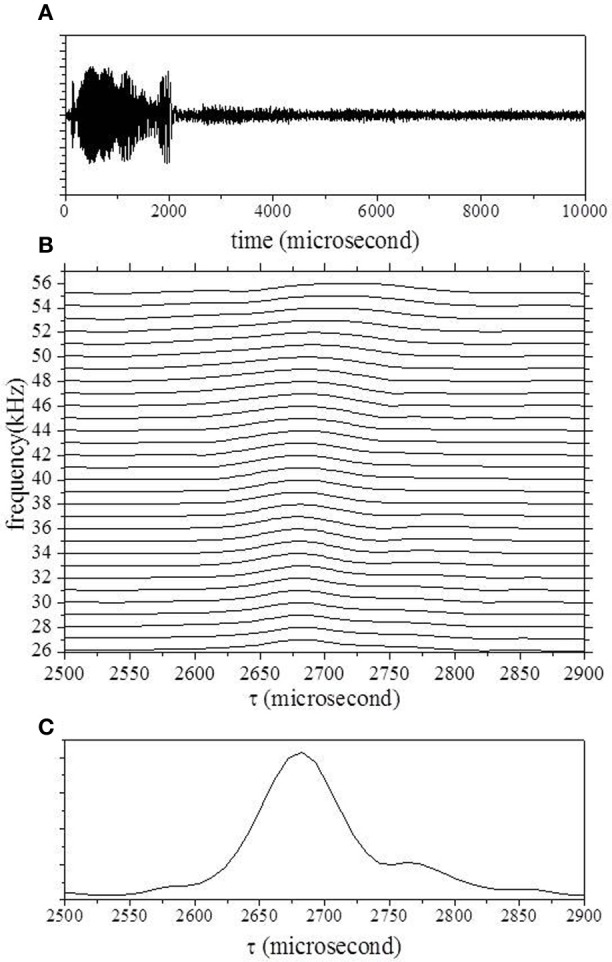
**(A)** Echo waveform. **(B)** Range–frequency pattern computed from the spectrogram, through convolution of the Gaussian chirplets. **(C)** Averaged pattern computed by integration of bandpass filter channels.

To demonstrate the output from the cochlear filters, we considered the situation of a static object with position (x, y) of (0 mm, 450 mm) and range of 901.8 mm. Figure 4A shows the measured waveforms including the object's echo as well as the sound transmitted from the loudspeaker. The spectrogram *P*(*f, t*), which was computed from the outputs of the Gaussian chirplets, was transformed into a range-frequency pattern *S*_echo_ (*f*, τ) with 10-μs intervals by compensating for the sweep rate, as shown in Figure 4B. The compensation time is denoted τ, and it is implied that the range corresponds to the delay time since the start time of emission was zero.

### Determination of the object's range and location in 2D space

**Figure 5:**
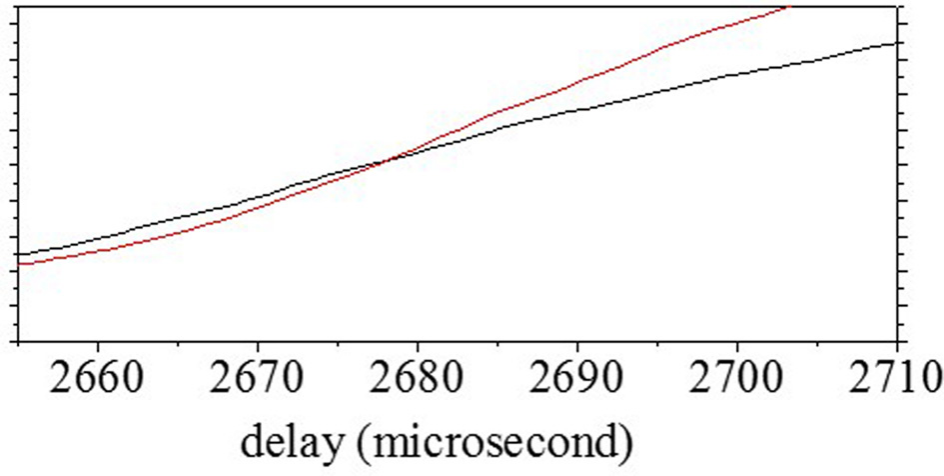
**Determination of *T*_1_**. The solid curve shows candidates for the reflected intensity distribution estimated from *S*_echo_ at onset delay time τ_on_. The dotted curve shows candidates for the reflected intensity distribution estimated from *S*_echo_ at 10 μs after τ_on_. *T*_1_ was determined by estimating the correspondence between the reflectivity of candidates at each delay timepoint.

The delay time for one object, *T*_1_, was estimated from the range–frequency pattern around the onset (Matsuo et al., 2004; Matsuo, 2011, 2013). First, the averaged pattern was computed by the integration of bandpass filter channels. The delay times for the onset and offset were determined using a threshold corresponding to almost four times the noise level (Matsuo, 2011, 2013). *T*_1_ and the corresponding reflectivity, *r*_1_, were uniquely determined from the averages of the two spectra at the onset delay time τ_on_ and 10 μs later. Figure 4C shows the averaged pattern, which was computed from the range–frequency pattern shown in Figure 4B. In this case, the delay time τ of the onset was estimated using a threshold of 2630 μs. The black curve in Figure 5 shows candidates for *T*_1_ according to the reflected intensity distribution estimated from the average of *S*_echo_ at the onset delay τ_on_ (2630 μs). The red curve shows candidates estimated from the average of *S*_echo_ 10 μs after τ_on_ (2640 μs). The delay time for *T*_1_ was determined to be 2678 μs, corresponding to 908.6 mm, by comparing the correspondence between the reflected intensities of the two candidates.

The location of the object in 2D space was determined by the difference between the object's ranges at the two microphones. The object's position (*x, y*) is represented by polar coordinates (*r*, θ):

$$\begin{array}{l} x=r\sin(\theta ),\\ y=r\cos(\theta ), \end{array}$$

where *r* is the distance between the speaker and the object, and θ the direction of the object with respect to the horizontal axis. The distance *r* between the speaker and object was obtained from the mean of the ranges at the two microphones. If *r* >> *d*, corresponding to the distance between two microphones, the direction of the object, θ, was computed from this difference, Δr, using the approximation

$$\theta =\sin^{-1}\left(\frac{\Delta r}{d}\right)$$

The object was continuously tracked by estimating its position at each timepoint.

## Results

### Localization of one pole moving back and forth

**Figure 6:**
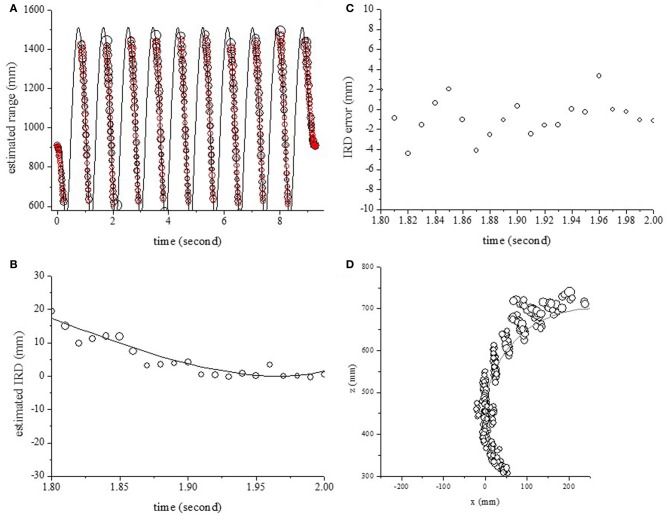
**Outputs for one object, moving back and forth with a rotation radius of 250 mm. (A)** Estimated and actual ranges. **(B)** Estimated IRDs. **(C)** Estimation errors of IRDs. **(D)** Estimated and actual positions in 2D space.

To evaluate the effect of the Doppler shift on the localization accuracy, the echoes from one object moving back and forth were measured and analyzed. The center of rotation was fixed at (250 mm, 450 mm) and the radius of rotation was 250 mm. The circles in Figure 6A show the estimated range along the time axis. The object's range could be estimated accurately using the temporal changes of echo spectra at the onset time. Figure 6B shows the IRD at each timepoint when the pole was moving back to forth. As shown in Figure 6C, the errors of the IRD were less than 4 mm. The circles and curves in Figure 6D show the estimated location and position of the object in 2D space. The locations of one pole could be estimated using the object's ranges for two microphones.

### Localization of one pole moving from side to side

**Figure 7:**
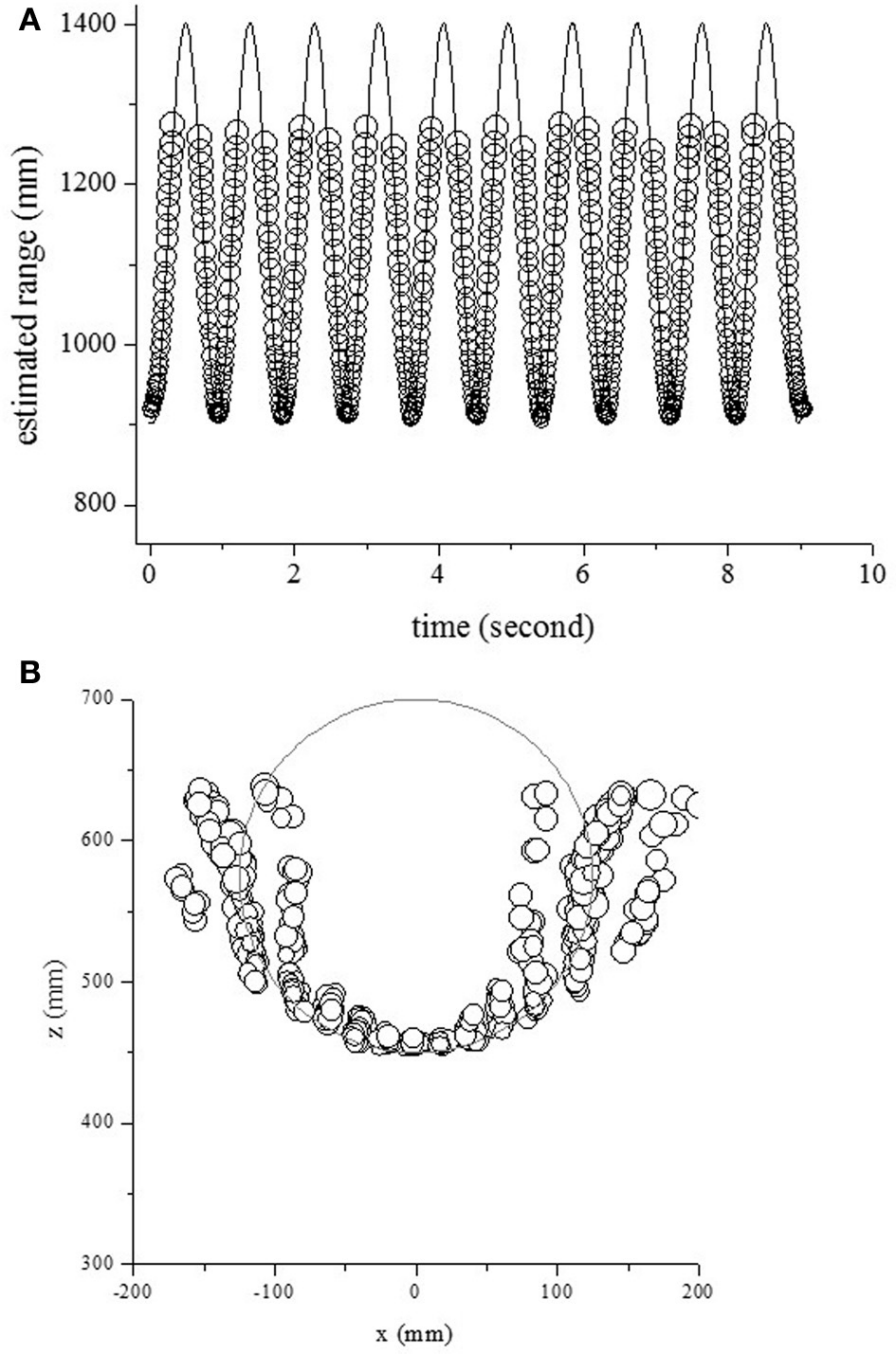
**Outputs for one object, moving side to side with a rotation radius of 125 mm. (A)** Estimated and actual ranges. **(B)** Estimated and actual positions in 2D space.

To evaluate the model's performance for different movements, the echoes from one pole moving from side to side were measured and analyzed. First, the center of rotation was fixed at (0 mm, 575 mm) and the radius of rotation was 125 mm. The circles and curves in Figure 7A show the estimated range and the object's actual range along the time axis. The object's range could be estimated using the temporal changes of echo spectra at the onset time. The circles and curves in Figure 7B show the estimated location and object's position in 2D space, respectively. One pole could be localized except for side positions.

**Figure 8:**
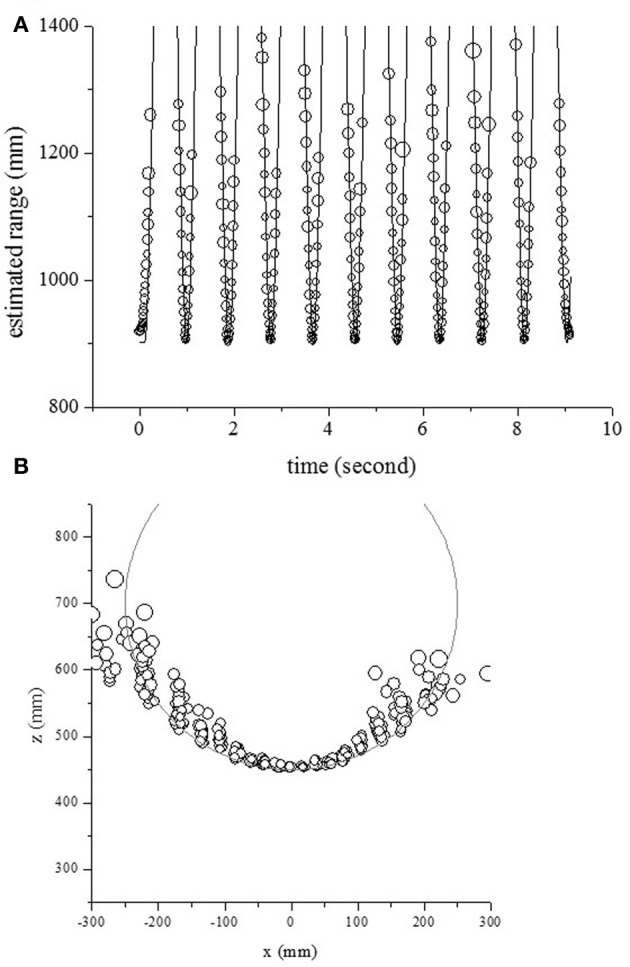
**Outputs for one object, moving from side to side with a rotation radius of 250 mm. (A)** Estimated and actual ranges. **(B)** Estimated and actual positions in 2D space.

In the second measurement scenario, the center of rotation was fixed at (0 mm, 700 mm) and the radius of rotation was 250 mm. The circles and curves in Figure 8A show the estimated range and the object's actual range along the time axis. The object's range could be estimated using the temporal changes of echo spectra at the onset time. The circles and curves in Figure 8B show the estimated location and object's position in 2D space, respectively. One pole could be localized except for side positions.

## Discussion and conclusion

Bats can locate and discriminate between individual objects even when the objects are moving (Griffin, 1958; Webster and Griffin, 1962; Griffin et al., 1965; Simmons et al., 1995). In a previous study, echoes were measured from a moving object while emitting (LFM) sound intermittently. The object's range and location in 2D space was estimated by extracting the temporal changes of echo spectra. In this paper, bat-like LPM sound was used to localize a moving object. It was demonstrated that this model could extend the localization of the moving object from echoes using the LPM signal. For this model, the errors in the IRD were less than 4 mm, corresponding to 12 μs, as shown in Figure 6, while the errors in the IRD using the LFM signal (Matsuo, 2013) were less than 2 mm, corresponding to 6 μs. The range accuracy was dependent on the signal-to-noise ratio (SNR) and the frequency bandwidth (Burdic, 1968; Menne and Hackbarth, 1986; Simmons et al., 2004; Boonman and Ostwald, 2007). The frequency bandwidths were 23 kHz in this model using the LPM signal, and 70 kHz in the previous model using the LFM signal. It is thought that the difference of errors is due to differences in frequency bandwidths of the emitted sound.

Bat can perceive the object in 3D space by localizing object's distance and direction. Directional information by real bats has previously been investigated by measuring the head-related transfer function (Wotton et al., 1995; Aytekin et al., 2004; Mey et al., 2008). Therefore, it is necessary to extend to localize objects in 3D space using the IRD in combination with the interaural level difference and the transfer function.

In this paper, only the first harmonics of the LPM signal were used. *Eptesicus fuscus* emits ultrasonic frequency modulation sounds containing two prominent downward-sweeping harmonics. In behavioral studies, echo-delay perception was disrupted by small temporal misalignments of echo harmonics (Bates and Simmons, 2011; Bates et al., 2011). Thus, the temporal cues for two harmonics are important to echolocation in nature. In future work, it will be necessary to extend this model to describe these results using harmonic sound signals.

### Conflict of interest statement

The author declares that the research was conducted in the absence of any commercial or financial relationships that could be construed as a potential conflict of interest.
